# Rates and predictors of service disengagement in adolescents with first episode psychosis: results from the 2-year follow-up of the Pr-EP program

**DOI:** 10.1007/s00787-023-02306-5

**Published:** 2023-10-09

**Authors:** Lorenzo Pelizza, Emanuela Leuci, Emanuela Quattrone, Silvia Azzali, Simona Pupo, Giuseppina Paulillo, Pietro Pellegrini, Marco Menchetti

**Affiliations:** 1 Department of Biomedical and Neuromotor Sciences, Alma Mater Studiorum Università di Bologna, Istituto di Psichiatria “Paolo Ottonello”, viale Pepoli 5, 40123 Bologna, BO Italy; 2 Department of Mental Health and Pathological Addictions, Azienda USL di Parma, largo Palli 1/a, 43100 Parma, PR Italy; 3 Department of Mental Health and Pathological Addictions, Azienda USL-IRCCS di Reggio Emilia, viale Amendola 2, 42100 Reggio Emilia, RE Italy; 4 Pain Therapy Service, Department of Medicine and Surgery, Azienda Ospedaliero-Universitaria di Parma, viale Gramsci 14, 43100 Parma, PR Italy

**Keywords:** Disengagement, Dropout, Early intervention in psychosis, First episode psychosis, Adolescent, Follow-up

## Abstract

Service disengagement is a major concern for “Early Intervention in Psychosis” (EIP). Indeed, understanding predictors of engagement is important for the effectiveness of mental health interventions, to improve outcome and quality of life, also in adolescents with first episode psychosis (FEP). No specific European investigation on this topic in adolescence has been reported in the literature to date. The aim of this study was to investigate service disengagement rate and predictors in an Italian sample of FEP adolescents treated within an EIP program during a 2-year follow-up period. All participants were adolescents help-seekers (aged 12–18 years) enrolled in the “Parma Early Psychosis” (Pr-EP) program. At baseline, they completed the Positive and Negative Syndrome Scale (PANSS) and the Global Assessment of Functioning (GAF) scale. Univariate and multivariate Cox regression analyses were performed. 71 FEP adolescents were recruited in this research. During the 2 years of our follow-up, a 25.4% prevalence rate of service disengagement was found. Particularly robust predictors of disengagement were lower baseline acceptance of psychosocial interventions, substance abuse at entry, and lower baseline PANSS “Disorganization” factor score. Approximately, 1/4 of our FEP adolescents disengaged from the Pr-EP program during the first 2 years of treatment. A possible solution to decrease disengagement and to favor re-engagement of these young individuals might be to provide the option of low-intensity monitoring and support, also via remote technology.

## Introduction

Service disengagement is a major concern that afflicts “Early Intervention in Psychosis” (EIP) programs, contributing to poor outcomes and high healthcare costs [1]. It was reported that the therapeutic benefits of EIP interventions are largely influenced by the degree to which subjects with first episode psychosis (FEP) engage in treatment, with relevant consequences in terms of risk of relapse and poor daily functioning [2]. A recent meta-analysis on the strength of engagement in EIP services found a 15% pooled prevalence rate of service disengagement despite ongoing therapeutic need, with high heterogeneity (1–41%) across studies [3]. These discordant results were mainly attributed to different definitions of engagement and variations in follow-up length [4]. Specifically, disengagement definitions varied from “when individuals actively refused any contact with the treatment staff” [5] to “terminated treatment despite therapeutic need” [6]. This definition discrepancy is also because engagement in treatment is a multidimensional and dynamic phenomenon that encompasses multiple factors (such as acceptance of a need for help, therapeutic alliance, mutual working toward shared goals, satisfaction with the therapy already received, differences in service models, longitudinal changes in patient needs) [4]. In this respect, based on their meta-analytic evidence, Robson and Greenwood [3] recently proposed a more coherent definition of service disengagement: i.e., a complete lack of contact or untraceable for 3 months despite a need for treatment, counted from the date of the last clinical contact. With this definition, the authors excluded FEP people who moved out from the catchment area, those who were “appropriately” discharge (i.e., after a relevant clinical improvement) and those who died or were imprisoned, on the basis that any conclusions about engagement could not be drawn from these events.

Another significant contributor to high sample heterogeneity is the variation in follow-up length, where shorter research may capture an artificially inflated disengagement rate including FEP patients who have temporarily dropped out. Indeed, the highest disengagement rate came from a study measuring disengagement at 9 months (41%), whereas a disengagement prevalence of 9.6% was from a 5-year follow-up investigation [3]. In this respect, most of 1-year disengagement rates seem to be related to FEP individuals disengaged and re-engaged through hospitalization or as outpatients [4].

Meta-analytic evidence also suggested that the most robust predictors of service disengagement in young FEP populations are poor medication adherence and substance abuse [3]. Moreover, other consistent findings came from studies also examining symptom severity and functioning, which found lower symptoms and higher global functioning as risk factors for disengagement [4]. Finally, some evidence involved the impact of family support and previous contact with the criminal justice system [3]. However, most of these investigations on risk factors and/or moderators of service disengagement were conducted on mixed adolescent and young adult FEP samples (i.e., participants aged 14–35 years). Therefore, future research specifically involving FEP adolescents is needed. In this respect, only Schimmelmann and co-workers in Australia examined predictors of disengagement in adolescents with FEP, but their study was a retrospective cohort investigation collecting data from clinical files [7]. According to Golay and co-workers [8], further studies (especially with longitudinal design) are needed to clarify these mixed results and to increase our knowledge on predictive factors that are most important for disengagement from EIP services, especially in adolescence.

Furthermore, it is well known that being adolescents is in itself a relevant risk factor for disengaging from mental health services (especially at the time of the adolescent–adult transition) [9]. This is of crucial importance for people with FEP, given that its peak onset often occurs during adolescence and outcome trajectories are established relatively early (usually during the first 2–3 years from presentation) [10–12]. In this respect, service disengagement is a major problem in early intervention for psychosis services [4]. Indeed, a crucial element is the willingness and ability of service users to engage in treatment, with those who disengage or are only superficially engaged that are at greater risk of relapse and poor prognosis [3].

Starting from this background, the aim of this investigation was to longitudinally assess disengagement rates and predictors in an Italian sample of FEP adolescents treated within a specialized EIP service across a 2-year follow-up period. As mentioned above, only one study on service disengagement in FEP adolescents has been reported in the literature to date [7]. It was conducted within the “Early Psychosis Prevention and Intervention Centre” (EPPIC) in Melbourne (Australia), but had a retrospective design (with data collected from clinical charts). To our knowledge, no European research exclusively on FEP adolescent population has been carried out.

## Methods

### Setting and subjects

All participants were FEP adolescents who entered the “Parma Early Psychosis” (Pr-EP) program from January 2013 to December 2020. The Pr-EP is a 2-year EIP protocol that was developed and implemented not as a centralized (stand-alone) service, but as a diffuse infrastructure in all adolescent community mental healthcare services of the Parma Departments of Mental Health, in Northern Italy [13]. FEP help-seeking patients were referred to the Pr-EP program mainly by primary care practitioner, general hospital (including emergency room), school and social services, family members, other generalist (first-line) mental healthcare services, or were self-referred. Specifically, based on symptom severity, the Pr-EP protocol offered a 2-year comprehensive treatment package including a psychopharmacological therapy and a multi-element psychosocial intervention that combined individual psychotherapy (mainly based on cognitive-behavioral principles), psychoeducational sessions for family members, and a recovery-oriented case management, in accordance with the current EIP guidelines [14, 15]. Low-dose atypical antipsychotic drug was used as first-line treatment [16]. Benzodiazepine and selective serotonin reuptake inhibitor could also be used in case of anxiety, depression, or insomnia [17]. Individual psychotherapy was developed on the cognitive–behavioral modules for psychotic disorders proposed by Garety and colleagues [18]. Family intervention was based on the cognitive–behavioral model for psychosis suggested by Falloon [19]. As for case management, each participant/family had a dedicated case manager offering an early recovery-oriented rehabilitation and coordinating all the planned interventions, especially those aimed at promoting job and social inclusion [20].

Inclusion criteria for this study: (a) age 12–18 years; (b) specialist help-seeking request; (c) enrollment within the Pr-EP program; (d) presence of FEP within one of the following DSM-5 diagnoses [21]: schizophrenia, bipolar disorder with psychotic features, major depressive disorder with psychotic features, delusional disorder, brief psychotic disorder, schizophreniform disorder, and psychotic disorder not otherwise specified; and (e) a duration of untreated psychosis (DUP) of < 2 years (the DUP was defined as the time interval [in months] between the onset of overt psychotic symptoms and the first antipsychotic intake) [22]. This DUP range was selected because it is the usual time limit for offering effective interventions within the EIP paradigm [23]. Information on DUP was collected directly by Pr-EP team members at the enrollment in the program, both consulting medical records and during appointments with patients and family members.

Exclusion criteria were: (a) past episode of DSM-5 affective or non-affective psychosis; (b) past exposure to antipsychotic drug; (c) known intellectual disability (i.e., IQ < 70); and (d) neurological disease or any other medical condition presenting with psychiatric symptoms. In this investigation, past exposure to antipsychotic medication (i.e., at any dosage and at any time before the Pr-EP enrollment) was considered as “functional equivalent” of past psychotic episode, in line with the psychometric criteria for psychosis threshold proposed in the current EIP paradigm (i.e., those for whom antipsychotic medication would probably be started in common clinical practice) [24]. In this sense, while aware that this exclusion criterion could limit most youth from participating in the study, we nevertheless excluded FEP adolescents with past exposure to antipsychotic medication to select individuals with shorter DUP who should then have responded better to Pr-EP treatments. The fulfillment of the inclusion and exclusion criteria was carefully assessed at baseline by trained Pr-EP team members.

### Measures

For the specific purpose of this study, a sociodemographic/clinical chart (collecting information on gender, age at entry, ethnic group, migrant status, years of education, occupation, civil and living condition, past specialist contact, previous hospitalization, current substance abuse, DUP, past suicide attempt, and acceptance of psychopharmacological and/or psychosocial interventions) was completed by Pr-EP team members at baseline (both consulting medical records and directly during appointments with patients and their family members). With the term “baseline”, we intended the time of recruitment into the Pr-EP program after the referral. Specifically, we defined “suicide attempt” as a potentially injurious, self-inflicted behavior without a fatal outcome for which there was (implicit or explicit) evidence of intent to die [25]. Moreover, the term “current substance abuse” referred to the harmful or hazardous use of psychoactive substances (including alcohol and illicit drugs), as well as a recurring desire to continue taking the drug despite harmful consequences [21]. Information on substance abuse was gathered at entry both in medical records and directly during clinical visits with patients and their family members). Finally, the acceptance of psychopharmacological and psychosocial treatments was evaluated within an individualized therapeutic-rehabilitation plan, which was shared and jointly signed by the patient, her/his family members, and mental health professionals [13].

The psychopathological assessment included the Positive and Negative Syndrome Scale [26], the Global Assessment of Functioning (GAF) scale [21] and the Health of the Nation Outcome Scales for Children and Adolescents (HoNOSCA) [27]. All these instruments were administered by trained Pr-EP team members at baseline. Regular supervision sessions and scoring workshops were used to ensure their inter-rater reliability [28]. These tools are part of standard care within the Pr-EP program.

The PANSS is a structured clinical interview developed to evaluate psychosis psychopathology. It has been commonly used also in young FEP populations [29]. As indicated by Shafer and Dazzi [30], we considered five main psychopathological dimensions: “Affect” (“Depression/Anxiety”), “Negative Symptoms”, “Positive Symptoms” “Disorganization” and “Resistance/Excitement–Activity”.

The GAF is a widely used scale for measuring daily functioning in patients with psychosis. It has been frequently administered also in young people with FEP [31].

The HoNOSCA was developed to assess social and clinical outcomes in children and adolescents with severe mental illness. It has been commonly used also in adolescents with FEP [32]. As proposed by Gowers and co-workers [33], we considered four main outcome domains: “Psychiatric Symptoms”, “Social Problems”, “Impairment” and “Behavioral Problems”. As indicated in the “Mental Health Clustering Tool” (MHCT) that is commonly used in UK clinical practice to measure patient well-being and to allocate service users to care clusters under the care pathway and packages approach [34], together with HoNOSCA current items, we also considered 5 “historical” scores on events that may remain relevant to the current plan of care. Among these historical ratings, there is an “Engagement” item specifically measuring patients’ treatment motivation, insight on their problems, acceptance of intervention proposals and ability to relate to the care staff. Specifically, high scores on this item reflect low levels of engagement and care motivation.

### Procedures

The *DSM*-5 diagnosis was formulated at entry by at least two trained Pr-EP team professionals using the Structured Clinical Interview for DSM-5 mental disorders (SCID-5) [35]. The presence of FEP was further confirmed using the psychometric criteria of the Comprehensive Assessment of At-Risk Mental States (CAARMS), authorized Italian version [36].

As proposed by Robson and Greenwood [3] in the only meta-analysis on engagement in EIP services published in the literature to date, we defined service disengagement (SD) as “complete lack of contact or untraceable for at least 3 months despite a need of treatment, counted from the date of the last face-to-face meeting with the clinical staff”. This comprehensive definition included FEP individuals “who actively refused further contact with the treatment team and were no longer traceable” [7], those “who did not return phone calls or did not attend appointments for at least 3 months despite therapeutic need” [37], and those “who prematurely exit EIP treatments against clinicians’ advice” [38]. Moreover, in line with meta-analytic suggestions, we excluded FEP participants who moved out of our catchment area, those who were appropriately discharged (i.e., who were clinically improved and subsequently transferred to other [private or public] generalist mental healthcare professionals), and those who died or were incarcerated, on the basis that any conclusions about engagement could not be drawn from these events. FEP patients meeting criteria for our definition of service disengagement were included in the FEP/SD + subgroup. The remaining participants were grouped in the FEP/SD− subsample.

For identifying predictive factors of service disengagement in the FEP total group, we finally investigated any significant association with functioning, sociodemographic and clinical characteristics at entry, as well as with baseline acceptance of specialized Pr-EP interventions.

### Statistical analysis

Data were analyzed using the Statistical Package for Social Science (SPSS) for Windows, version 15.0 [39]. Statistical analyses were two-tailed with a significance level set at 0.05.

Cumulative proportional risk rates of service disengagement were investigated using the Kaplan–Meier survival analysis, which is able to take into account the time of survival (in months) among the FEP participants entered the 2-year follow-up period [40].

For identifying any relevant predictive factor of service disengagement in our population, statistically significant associations of disengagement with baseline acceptance of Pr-EP therapeutic proposals and sociodemographic and clinical characteristics at entry were examined in the FEP total sample using Cox regression analyses. Due to multiple comparisons, we used the Holm–Bonferroni method for *p* value correction. After having previously checked that the proportionality-of-hazards assumption was met [41], univariate models were fitted for each potential predictor of service disengagement. As for potential predictors, we used an exploratory approach considering all baseline sociodemographic and clinical variables collected in our “ad hoc” sociodemographic/clinical chart, also including information on acceptance of Pr-EP intervention proposal at entry. The predictive parameters that resulted statistically relevant were then put as covariates into a multivariate Cox regression analysis to test the strongest predictive parameters for service disengagement in our adolescent FEP population. This two-step method allowed us to adapt the number of covariates to the size of our FEP sample, keeping a ratio equal to at least 1:20 (i.e., 20 participants for each covariate) [42]. Finally, we performed receiver operating characteristic (ROC) curve analysis for 2-year service disengagement using the predictive factors resulted statistically significant in the Cox regression models as test variables and potential classifiers for service disengagement prediction.

## Results

**Fig. 1:**
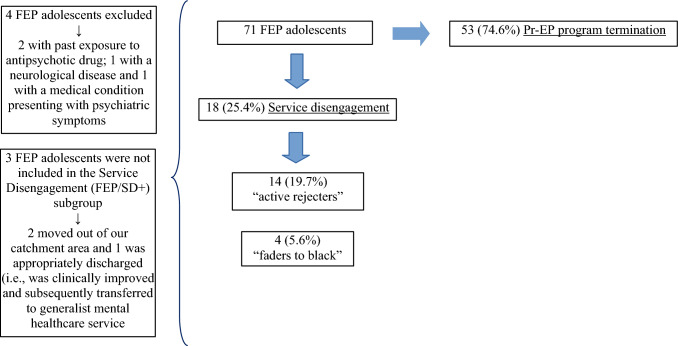
Prevalence rates of service disengagement across a 2-year follow-up period in the FEP total sample (*n* = 71)

A total of 71 FEP adolescents were recruited in this investigation (4 patients enrolled in the Pr-EP program were not included due to exclusion criteria) (Fig. 1). 18 (25.4%) of them disengaged from the Pr-EP program and were included in the FEP/SD + subgroup. The remaining 53 individuals concluded the 2-year follow-up period and were grouped in the FEP/SD- subsample. Finally, three FEP participants were not included in the Service Disengagement (FEP/SD +) subgroup due to our definition of service disengagement (i.e., 2 moved out of our catchment area and 1 was appropriately discharged and subsequently transferred to generalist mental healthcare service).

Among “disengagers”, 14 actively refused contact with the treatment staff and were no longer traceable against clinician’s advice (“active rejecters”), and only 4 simply did not return phone calls or did not attend appointments for at least 3 months, despite ongoing therapeutic need (i.e., they did not explicitly refused treatment, but silently dropped out of the Pr-EP program without being traceable any longer) (“faders to black”).

**Fig. 2:**
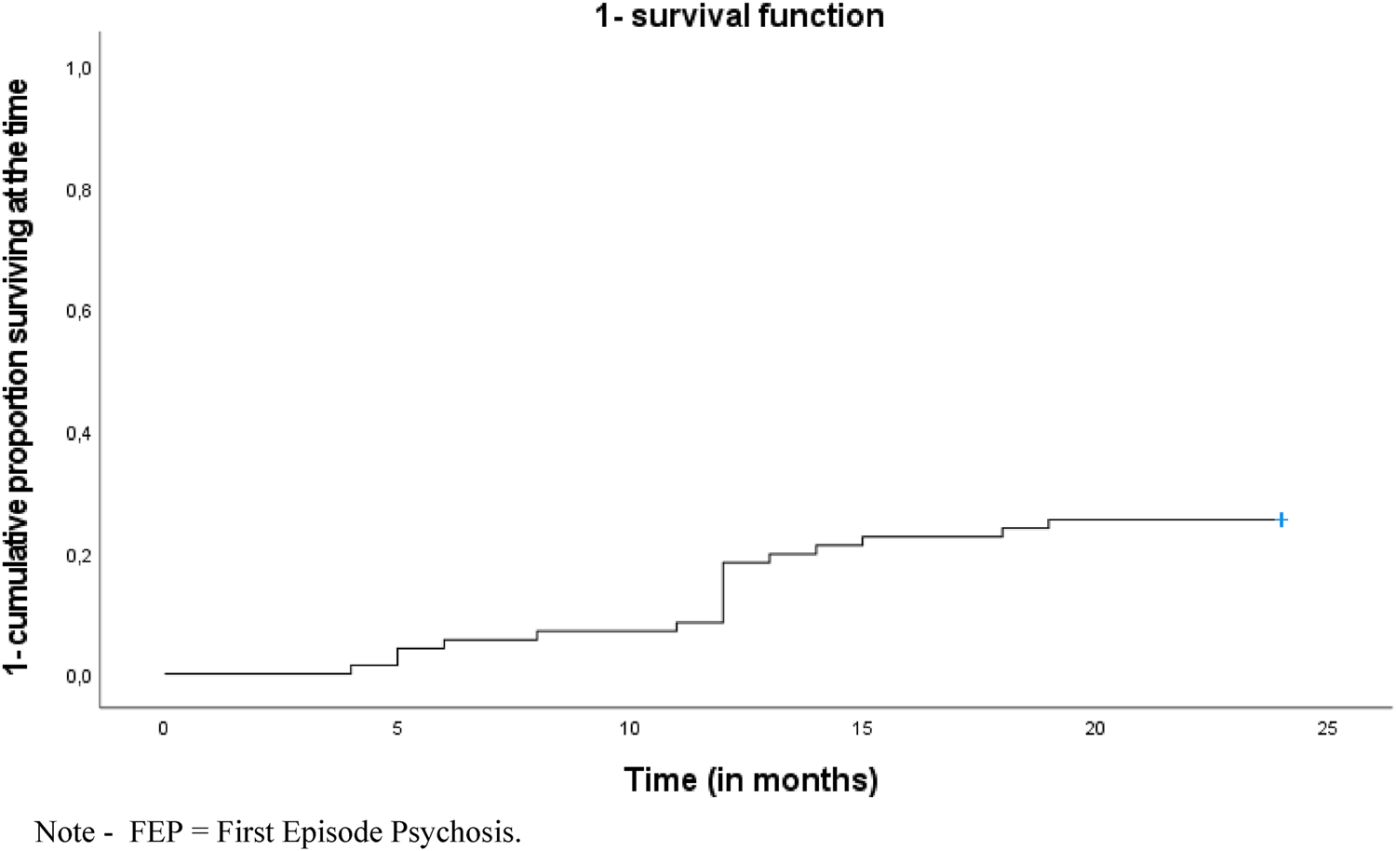
Kaplan–Meier survival analysis results on service disengagement across the 2-year follow-up period in the FEP total sample (*n* = 71): 1-survival function. *FEP* first episode psychosis

Kaplan–Meier survival analysis results confirmed a 2-year estimated cumulative service disengagement rate of 0.254 (Table 1 and Fig. 2). In the FEP total group, the DSM-5 diagnoses at baseline were schizophrenia (*n* = 34; 47.9%), affective psychosis (*n* = 24; 33.8%), brief psychotic disorder (*n* = 10; 14.1%), and psychotic disorder not otherwise specified (*n* = 3; 4.2%).

**Table 1:** Kaplan–Meier survival analysis results on service disengagement across the 2-year follow-up period in the FEP total sample (*n* = 71)

Variable	1-cumulative proportion surviving at the time	
	Estimate	SE
1-year service disengagement rate	0.183	0.046
2-year service disengagement rate	0.254	0.052

*FEP* first episode psychosis; “active rejecters” = FEP participants who actively refused contact with the treatment staff and were not traceable for at least 3 months; “faders to black” = FEP participants who did not return phone calls or did not attend appointments for at least 3 months, despite ongoing therapeutic need (i.e., they did not explicitly refused treatment, but silently dropped out of the Pr-EP program without being traceable any longer). Estimate and standard error (SE) values are reported

In the FEP total sample, 2-year service disengagement from the Pr-EP program was significantly predicted by substance abuse at entry, lower baseline acceptance of psychosocial interventions (i.e., individual psychotherapy, family psychoeducation or case management), and higher baseline MHCT “Engagement (historical)” item subscore (Table 2). Statistical trends for prediction of 2-year service disengagement (i.e., 0.05 < *p* < 0.01) were also found for female gender and lower baseline PANSS “Disorganization” factor score.

**Table 2:** Univariate Cox proportional-hazard models for 2-year service disengagement in the FEP total sample (*n* = 71)

Variable	FEP/SD + (*n* = 18)	FEP/SD− (*n* = 53)	Statistic test			
			HR	95% IC	*p*	*D*
Gender (female)	14 (77.8%)	27 (50.9%)	2.977	0.979–4.376	0.054	–
Ethnic group (White Caucasians)	16 (88.9%)	46 (86.8%)	0.884	0.230–3.846	0.870	–
Migrant status	2 (11.8%)	4 (7.5%)	0.685	0.157–2.982	0.614	–
Age (at entry)	16.94 ± 1.21	16.08 ± 1.35	0.948	0.686–1.311	0.747	–
Education (in years)	10.83 ± 1.25	10.25 ± 1.53	0.954	0.705–1.292	0.761	–
Civil status						
Single	11 (61.1%)	26 (49.1%)	0.612	0.237–1.580	0.311	–
Partnership	7 (38.9%)	27 (50.9%)	0.788	0.301–1.201	0.489	–
Living status						
Living with parents	12 (66.7%)	38 (71.7%)	0.544	0.247–0.888	0.744	–
Other cohabitations	6 (33.3%)	15 (28.3%)	0.124	0.099–0.211	0.877	–
Occupation						
Unemployed	3 (16.7%)	7 (13.2%)	0.678	0.195–2.358	0.541	–
Employed	1 (5.6%)	0 (0.0%)	0.172	0.022–1.342	0.093	–
Student	14 (77.8%)	46 (86.8%)	0.254	0.026–2.497	0.240	–
DUP (in months)	8.21 ± 6.89	10.91 ± 10.98	0.974	0.924–1.027	0.326	–
Previous hospitalization	5 (27.8%)	15 (28.3%)	1.003	0.358–2.815	0.995	–
Previous specialist contact	8 (44.4%)	27 (50.9%)	1.241	0.490–3.146	0.648	–
Previous suicide attempt	0 (0.0%)	4 (7.5%)	22.194	0.005–98.062	0.470	–
Substance abuse at entry	10 (55.6%)	15 (28.3%)	0.391	0.154–0.993	**0.019**	0.248
Baseline AP prescription	15 (83.3%)	42 (79.2%)	0.794	0.230–2.742	0.715	–
Baseline LAI-AP prescription	0 (0.0%)	1 (1.9%)	20.577	0.001–316.59	0.720	–
Baseline AD prescription	2 (11.1%)	10 (18.9%)	1.682	0.387–7.317	0.488	–
Baseline MS prescription	3 (16.7%)	3 (5.7%)	0.398	0.115–1.379	0.146	–
Baseline BDZ prescription	7 (38.9%)	12 (22.6%)	0.567	0.219–1.464	0.241	–
Baseline acceptance of individual psychotherapy	8 (44.4%)	48 (90.6%)	7.435	2.916–18.956	**0.0001**	0.492
Baseline acceptance of family psychoeducation	8 (44.4%)	45 (84.9%)	5.239	2.061–13.317	**0.0001**	0.405
Baseline acceptance of case management	9 (50.0%)	41 (77.4%)	3.151	1.249–7.953	**0.015**	0.261
Baseline DSM-5 diagnosis						
Schizophrenia	7 (38.9%)	27 (50.9%)	1.570	0.608–4.050	0.351	–
Affective psychosis	6 (33.3%)	18 (34.0%)	1.088	0.408–2.900	0.866	–
Brief psychotic disorder	4 (22.2%)	6 (11.3%)	0.446	0.147–1.356	0.155	–
Psychotic disorder NOS	1 (5.6%)	2 (3.8%)	0.613	0.081–4.608	0.634	–
PANSS						
Positive symptoms	18.18 ± 4.51	17.89 ± 5.53	1.011	0.903–1.131	0.851	–
Negative symptoms	23.55 ± 11.32	23.37 ± 7.68	1.004	0.933–1.079	0.924	–
Disorganization	15.27 ± 5.93	20.21 ± 7.33	0.911	0.824–1.008	0.070	–
Affect	15.09 ± 5.07	15.69 ± 5.49	0.980	0.876–1.095	0.719	–
Resistance/excitement–activity	8.27 ± 4.34	8.91 ± 4.11	0.965	0.829–1.122	0.641	–
PANSS total score	83.36 ± 23.13	89.59 ± 21.45	0.988	0.961–1.017	0.417	–
G12 lack of judgment/insight	3.00 ± 1.09	3.37 ± 1.70	0.882	0.598–1.301	0.527	–
GAF	45.58 ± 9.01	44.09 ± 11.57	1.011	0.958–1.068	0.684	–
HoNOSCA						
Behavioral problems	4.11 ± 3.77	3.28 ± 2.08	1.115	0.931–1.337	0.238	–
Impairment	2.44 ± 1.04	3.00 ± 2.04	0.876	0.682–1.124	0.298	–
Psychiatric symptoms	10.72 ± 3.30	10.53 ± 3.35	1.020	0.885–1.174	0.788	–
Social problems	6.61 ± 2.45	6.79 ± 3.69	0.986	0.862–1.127	0.832	–
Total score	23.89 ± 6.64	23.60 ± 7.81	1.004	0.945–1.066	0.989	–
MHCT “Engagement (historical)” item	1.17 ± 1.42	0.42 ± 0.79	1.586	1.129–2.229	**0.008**	0.240

*FEP* First episode psychosis; *SD* service disengagement; *FEP/SD *+ FEP patients with SD; *FEP/S−* FEP patients without SD; *DUP* duration of untreated psychosis; *AP* antipsychotic; *LAI-AP* long-acting injectable antipsychotic formulation; *AD* antidepressant; *MS* mood stabilizer; *BDZ* benzodiazepine; *DSM-5* Diagnostic and Statistical Manual of Mental Disorders-5th edition; *PANSS* Positive and Negative Syndrome Scale; *GAF* Global Assessment of Functioning; *HoNOSCA* Health of the Nation Outcome Scales for Children And Adolescents; *MHCT* Mental Health Clustering Tool; *HR* hazard ratio; *95% CI* 95% confidence intervals for HR; *p* statistical significance; *D* effect size

Holm–Bonferroni corrected *p* values are reported. Frequencies (and percentages) and mean ± standard deviation are reported. Statistically significant *p* values are in bold

Multivariate Cox regression analysis results showed that only lower baseline acceptance of psychosocial interventions was a statistically robust predictor of service disengagement from the Pr-EP program (Table 3).

**Table 3:** Multivariate Cox proportional-hazards models for 2-year service disengagement in the FEP total sample (*n* = 71)model 1

Variable	*B*	SE	Wald	df	*p*	HR	95% CI for HR	
							Lower	Upper
Substance abuse at entry	0.702	0.506	1.923	1	0.166	0.495	0.184	1.337
Baseline acceptance of psychosocial interventions	3.079	0.692	19.801	1	**0.0001**	21.745	5.602	84.412
MHCT “Engagement (historical)” item subscore	− 0.227	0.220	1.066	1	0.302	0.797	0.518	1.227

Overall (score): *χ*^2^ = 53.003; df = 3; *p* = 0.0001

*FEP* first episode psychosis; *Baseline acceptance of psychosocial interventions* baseline acceptance of at least individual psychotherapy, family psychoeducation or case management; *MHCT* Mental Health Clustering Tool; *B* regression coefficient; *SE* standard error, *Wald* Wald statistic value; *df* degrees of freedom; *p* statistical significance; *HR* hazard ratio; *95% CI* 95% confidence intervals for HR

Holm–Bonferroni corrected *p* values are reported. Statistically significant *p* values are in bold

When we included parameters having statistical trends (0.05 < *p* < 0.01) in univariate Cox regression analyses, also lower baseline PANSS “Disorganization” factor score showed a relevant role in the prediction of 2-year service disengagement from the Pr-EP protocol (Table 4). The results of our ROC curve analyses are shown in the Table 5 and overall confirmed Cox regression findings. Specifically, AUC values showed that baseline acceptance of psychosocial interventions was the best classifier in both prediction models of service disengagement of our FEP adolescents from the Pr-EP protocol.

**Table 4:** Multivariate Cox proportional-hazard models for 2-year service disengagement condition in the FEP total sample (*n* = 71), including parameters with statistical trends (0.05 < *p* values < 0.01) in univariate Cox proportional-hazard models (see Table 3)—model 2

Variable	*B*	SE	Wald	df	*p*	HR	95% CI for HR	
							Lower	Upper
Substance abuse at entry	1.113	1.018	1.196	1	0.274	3.044	0.414	22.365
Baseline acceptance of psychosocial interventions	4.038	1.113	13.165	1	**0.001**	56.688	6.402	501.984
MHCT “Engagement (historical)” item subscore	− 0.292	0.302	0.938	1	0.333	0.746	0.413	1.349
Gender (female)	− 0.133	0.735	0.033	1	0.856	0.875	0.207	3.694
PANSS “Disorganization” factor score	− 0.127	0.061	4.366	1	**0.017**	0.881	0.782	0.992

Overall (score): *χ*^2^ = 35.495, df = 5, *p* = 0.0001

*FEP* first episode psychosis; *Baseline acceptance of psychosocial interventions* baseline acceptance of at least individual psychotherapy, family psychoeducation or case management; *MHCT* Mental Health Clustering Tool; *PANSS* Positive and Negative Syndrome Scale; *B* regression coefficient; *SE* standard error, *Wald* Wald statistic value; *df* degrees of freedom; *p* statistical significance; *HR* hazard ratio; *95% CI* 95% confidence intervals for HR

Holm–Bonferroni corrected *p* values are reported. Statistically significant *p* values are in bold

**Table 5:** Receiver operating characteristic (ROC) curve results for 2-year service disengagement with the statistically significant predictive factors (*n* = 71)

	AUC	SE	*p*	95% CI	
				Lower	Upper
Variable (model 1)					
Substance abuse at entry	0.364	0.078	0.086	0.211	0.517
Baseline acceptance of psychosocial interventions	0.713	0.081	**0.007**	0.554	0.871
MHCT “Engagement (historical)” item subscore	0.645	0.082	0.067	0.485	0.805
Variable (model 2)					
Substance abuse at entry	0.434	0.102	0.518	0.235	0.634
Baseline acceptance of psychosocial interventions	0.758	0.099	**0.011**	0.564	0.952
MHCT “Engagement (historical)” item subscore	0.666	0.103	0.102	0.465	0.867
Gender (female)	0.583	0.099	0.413	0.389	0.777
PANSS “Disorganization” factor score	0.295	0.088	**0.043**	0.122	0.469

*AUC* area under the curve; *SE* standard error; *p* statistical significance; *95% CI* 95% confidence intervals for AUC; *MHCT* Mental Health Clustering Tool; *PANSS* Positive and Negative Syndrome Scale

Holm–Bonferroni corrected *p* values are reported. Statistically significant *p* values are in bold

## Discussion

The results of this investigation showed a 2-year service disengagement rate of about 25%. This finding is higher than what (15.6%) was reported as pooled prevalence in a recent meta-analysis on a large cohort of 6800 FEP patients including both adolescents and adults (age range = 14–64 years) [3]. However, it is similar to the disengagement rate (23.4%) observed in a FEP sample exclusively composed of adolescent help seekers recruited within the EPPIC service [12].

Meta-analytic evidence also found that heterogeneity across studies was very high, with disengagement rates ranging from 1 to 41%. Multiple moderators of disengagement have been called into question to understand these conflicting results [43].

One reason may be the lack of a universally accepted definition of disengagement across investigations. Indeed, it varied broadly from “FEP people not in treatment at the end of the study” [44] to “FEP participants completing psychiatric care despite ongoing therapeutic need and untraceable sometimes with a time limit of 3 months” [45]. Therefore, it is imperative to use more cohesive methodologies across studies so that clinical comparisons can be made more accurately. Based on their meta-analytic evidence, Robson and Greenwood [3] recently proposed a more coherent definition of service disengagement that we decided to adopt in this research: i.e., a complete lack of contact or untraceable for 3 months despite a need for treatment, counted from the date of the last clinical contact. As suggested by the same authors, we excluded FEP people who moved out of our catchment area, those who were “appropriately” discharge (i.e., with a relevant clinical improvement and subsequently transferred to other [private or public] generalist mental health professionals) and those who died or were imprisoned, on the basis that any conclusions about engagement could not be drawn from these events. Furthermore, we proposed to dichotomize “disengages” in patients who actively refused contact with the treatment staff (“active rejecters”) and subjects who simply did not return phone calls or did not attend appointments despite ongoing therapeutic need. The latter subgroup silently abandoned psychiatric care, leaving EIP services too early without manifesting an explicit treatment refusal (“faders to black”). In our opinion, this distinction is crucial because it makes it possible to detect a FEP group (i.e., faders to black) on which to focus additional care resources (e.g., home visits, regular contact) and a dedicated staff to avoid service disengagement. However, among FEP patients that disengage or “fade to black”, there probably also are individuals who are feeling better and/or feel like they no longer require services. Some authors referred to this concept as “positive disengagement” [46].

Other authors suggested using clinician-rated service engagement scales (such as the “Singh-O’Brien Level of Engagement Scale” [SOLES] or the “Service Engagement Scale” [SES]) [47, 48]. In line with their suggestions, the findings of our research showed a significant relationship between service disengagement measured as dichotomous variable (i.e., presence vs. absence) and the baseline MHCT “Engagement [historical]” item subscore, a continuous parameter of service engagement specifically included in the MHCT to measure patient well-being and to allocate service users to care clusters under the care pathway and packages approach [34].

Another relevant contributor to high sample heterogeneity is the wide variation in follow-up length, where shorter studies may capture an artificially inflated disengagement rate including FEP participants who have temporarily dropped out. In this respect, the highest disengagement rate came from a study that measured disengagement at 9 months (41%) [2], whereas a 9.6% disengagement prevalence was from a 5-year EIP program [49]. Moreover, more than half of the service disengagement seems to be related to FEP subjects disengaged and re-engaged through hospitalization or as outpatients [4]. Based on these findings, Robson and Greenwood [3] stated that investigations should be at least 18 months in duration to avoid increased disengagement rates created by capturing FEP patients who may have only temporarily disengaged. For the purpose of this investigation, a 2-year follow-up period was selected. This time range is longer than that was considered in the above-mentioned meta-analysis on engagement in FEP people (i.e., 15-month median time to disengage) and may partially explain the difference observed in prevalence rates. However, future research examining FEP people who reengage in EIP services is needed.

Finally, other mediators contributing to heterogeneity across investigations may be related to cultural factors (e.g., belonging to ethnic minorities) and variations in mental health service models (e.g., how different EIP programs operate, the kind of interventions they provide, the diversity in catchment populations they served) [4]. However, information on specific contextual socio-demographic variables (such as marginalization, violence and neighborhood poverty) was generally not reported.

### Predictors of service disengagement

The results of this research showed that a lower baseline acceptance of psychosocial interventions was the most robust predictor of service disengagement for FEP adolescents entered the Pr-EP program. This finding further supports the meta-analytic evidence on poor treatment adherence as consistent predictive factor related to leaving EIP services in mixed adolescent and young adult FEP populations [3]. It may reflect a low trust in the specialized care that EIP protocols provide to the patient and/or family members, or misconceptions about the therapeutic models offered by some service providers [50]. In this respect, qualitative studies reported possible frictions between the subjective meaning that patient gives to psychotic experiences and the promotion of specific interventions form a biomedical perspective (“service mismatch”) [51]. Moreover, treatment adherence needs to be understood also within a framework of shared decision making, in which patients, family member,s and EIP staff should find the balance between “the duty to care” and “the dignity of risk” (i.e., the right to make choice, to fail, and to learn). This typically structures an “aimless engagement” with a lack of shared purpose [4]. Finally, there could be a “reactive disengagement” in response to individual circumstances (such as the appearance of medication side effects or a quick returning to school or work). In the last case, engagement with EIP service becomes a second priority that FEP adolescents would follow through with if it does not impact on their primary priority [52]. However, it is also important to acknowledge that the age range of our FEP participants was lower than early psychosis studies in other countries (such as the North American context). This could affect the role that family involvement plays in engagement in our EIP service.

However, the results of this study seem to suggest that poor treatment adherence already is at baseline a clinical characteristic of those FEP adolescents who will subsequently drop out of the Pr-EP protocol. This was confirmed by the higher baseline MHCT “Engagement (historical)” item subscore, reflecting lower levels of care motivation and suggesting a possible role of less intensive efforts in EIP services for those FEP subjects considered to be highly likely to disengage. Identifying and implementing appropriate strategies to improve care motivation, to reduce disengagement or to re-engage FEP adolescents with no desire to engage and treatment non-adherence (also through remote technologies and text messaging) are therefore needed [53]. Given the young age of FEP patients, providing interventions based on patient’s unmet needs and aspirations are also crucial [54].

Another predictor of service disengagement from the Pr-EP program was substance abuse at entry. This finding supports meta-analytic evidence on substance misuse as one of the most robust predictive factors of leaving EIP programs observed in mixed adolescent and young adult FEP samples. Based on our results, this seems to be of particularly importance in FEP adolescents, suggesting the need to better understand engagement patterns in EIP protocols for people with comorbid FEP and substance use disorder [55].

An additional predictor of service disengagement from the Pr-EP program was the presence of lower severity levels in disorganization at entry. This is in line with consistent findings from empirical studies examining symptom severity and functioning, which reported lower symptoms and higher global functioning as risk factors for disengagement [3]. Moreover, it also could be that being less severe, they may actually do better and therefore stop with the EIP service. This suggests a possible subjective perception of a lower severity of their clinical picture in those FEP adolescents at risk for disengagement, as well as a conviction of a reduced need for treatment, so that attendance takes a lower priority than work, education or leisure activities [4]. With recent advances in digital technologies, incorporating models of remote or blended delivery could promote engagement on a more convenient and casual basis for these FEP adolescents, preventing complete discharge from mental healthcare services [56].

### Limitations

A first limitation was the lack of an international consensus on disengagement definition, which limits comparisons across investigations and hampers to reach generalizable conclusions. Although we used a coherent, evidence-based definition of engagement [3], it is imperative to implement more cohesive methodologies across investigations. In this respect, Mascayano and co-workers [4] suggested bringing key stakeholders together (e.g., through partnerships) to reach a universal consensus, to develop common measures of treatment disengagement and to design strategies for increasing the strength of engagement. This discussion should include mental healthcare professionals as well as FEP adolescents and their family members, considering that they may have different options and perspectives [57].

Second, our research did not account for “true non-engagers” (i.e., those FEP patients who refused any contact with the service from the start). Moreover, in line with the definition of service disengagement proposed by Robson and Greenwood [3], it should also be emphasized that our results did not consider FEP adolescents who moved out of our catchment area and who was appropriately discharged and subsequently transferred to generalist mental healthcare service.

Another weakness was the relatively small sample size (especially in the FEP/SD+ subgroup). This is of relevance and may bring our results into question, especially considering the number of covariates in regression models. Therefore, further research on larger FEP adolescent populations to replicate our results is needed. However, power analyses for univariate Cox regression models were performed to appreciate type II errors (see the Table 2 for details). The power of the tests was calculated in accordance with what was proposed by Schoenfeld [58] using the R package “powerSurvEpi” [59].

Finally, this investigation was limited to a 2-year follow-up period. Our findings were thus comparable exclusively with longitudinally similar studies.

## Conclusions

The results of this investigation showed that about 25% of FEP adolescents enrolled in the Pr-EP program dropped out during the first 2 years of treatment. Particularly robust predictors of service disengagement were baseline non-acceptance of psychosocial interventions and substance abuse at entry. There is also evidence that FEP adolescents with lower baseline severity levels in disorganization were more vulnerable to disengagement. For these individuals, a solution might be to remain on EIP program caseloads, allowing the option for low-intensity support and monitoring, also via remote technology.

## Data Availability

The data that support the findings of this investigation are available on reasonable request from the corresponding author. The data are not publicly available due to privacy/ethical restrictions.
